# Salubrol, a Substitute for Iodoform

**Published:** 1896-09

**Authors:** Paul De Terra

**Affiliations:** Dentist, Zürich, Switzerland


### Abstracts and Translations.

### SALUBROL, A SUBSTITUTE FOR IODOFORM.

BY PAUL DE TERRA, DENTIST, ZÜRICH, SWITZERLAND.

In surgery nowadays carbolic acid, corrosive sublimate, and iodoform are still predominant, and in dentistry these antiseptics, although not of equal importance, have likewise the preference. In the treatment of the teeth, with especial reference to the pulp, we require two principal specifics: one for the treatment of the pulp itself when it is to be preserved, and a second for the root-canals when the pulp has been destroyed. In both cases an antiseptic with corrosive action is thoroughly impracticable. Especially for the antiseptic treatment of roots, carbolic acid and mercuric chloride seem to me extremely dangerous, particularly if these powerful agents have not been used with the utmost caution, and, for example, they have penetrated into the alveolar process through the foramen apicale, through defective canal walls, or by a simple process of absorption. How many cases of pericementitis have been the consequence of this disastrous treatment with these agents, to say nothing of the great possibility of necrosis, and the consequent loss of the tooth or even of the entire alveolar process?

The only specific which we have heretofore been able to use in all cases of pulpitis and pericementitis, without danger to the delicate tissues, is iodoform. The latter has, as is well known, no corrosive action, and is therefore an invaluable antiseptic, especially for the treatment of roots. But that iodoform, in spite of its antiseptic qualities, has no effect as a germicide has been established by the investigations of Miller and others, and, indeed, soon after the discovery of this agent, which at the time was regarded as making an epoch in antiseptic treatment, the inadequacy of iodoform was pointed out at the Medico-Chirurgical Society of London in 1882.

That, in spite of this, iodoform still holds the first place in general estimation is proved by the fact that it continues to be an important factor in the filling of roots, and that surgery is still seeking in vain for an equivalent which shall possess the desirable qualities for treating wounds by this excellent agent, and at the same time avoid its defects. Especially noteworthy among the latter are its penetrating odor, its incidental toxic effect, and its imperfect efficiency as an antiseptic.

By the repulsive odor of iodoform the use of this otherwise serviceable agent is considerably limited in practice, especially for us dentists. Accordingly, since its first production, different correctives have been suggested with a view to deodorizing the preparation (ether, oils, sassafras, Peru balsam, and particularly Cumarin), and, in addition to these, a substitute for iodoform has been devised in iodol. Aside from the fact that none of these admixtures materially improve the odor of iodoform, they have, in part at least, a tendency to diminish its antiseptic value, and even to make its use in surgery in this form almost impossible.

The second disadvantage of iodoform, its incidental toxic effect, is of more importance for medicine and surgery, for in dentistry there can generally be no question of any such danger of poisoning, since the quantity of iodoform employed at any one time is in reality too small. Nevertheless, it must not be forgotten that the action of this drug varies greatly with the patient. The most dangerous properties of iodoform depend upon the fact that iodine, which is its principal constituent, and which makes up ninety-six per cent. of the preparation, is often not thrown off from the organism for a considerable period, and in this way induces a much-dreaded cumulative effect. The urine of patients who have been treated with iodoform may show appropriate reaction for weeks without any fresh application of the drug.

In view of these three important defects of iodoform, it would seem that an antiseptic which, while similar to iodoform in all its good qualities, is free from these disadvantages, and which at the same time possesses a greater antiseptic power, might not only prove a satisfactory substitute for iodoform, but might even be destined to supplant it in practice.

Such an antiseptic seems now to have been found. A Zürich chemist, Dr. Schuftan, some time ago produced a preparation which fully satisfies the above-mentioned conditions, and which has been placed upon the market under the name of salubrol by the Farbwerken, in Höchst, near Frankfurt-am-Main. The preparation is produced by the action of bromine upon methylbisantipyrin, and its chemical composition corresponds approximately to that of a tetrabromide. The bromine in it is very loosely united, and this characteristic renders all attempts to recrystallize the compound unavailing. It must be remarked, however, that the preparation itself is quite stable, and that it is only when in contact with organic substances that it gradually gives off a part of its bromine. It is probable that the efficiency of salubrol as a germicide may be traced to this very circumstance, for the antiseptic property of iodoform also is explained by the fact that the iodine contained in it belongs to the halogen group, all the members of which have a strong affinity for hydrogen, so that the liberated halogen unites with the hydrogen of water contained in the tissues, while the oxygen thus set free oxidizes the septic mass.

Salubrol is an extremely fine powder of a light (sulphur) yellow tint, which in taste and smell suggests the pharmacy; it is insoluble in ether and in water, but easily soluble in alcohol and chloroform. In water, and especially in glycerin, salubrol remains intimately suspended for a long time, so that it is very well adapted for injections and pastes. In addition to being free from offensive odor, salubrol has the still more important advantage over iodoform that it is not poisonous. This fact has been demonstrated by repeated tests made with animals, and in the different hospitals and dermatological clinics of Germany and Switzerland, by its use in the private practice of many specialists, and by the very conscientious experiments of Dr. Silber, in Breslau.[1] Moreover, when we consider the chemical composition of salubrol it is obvious that a toxic effect on the part of this drug would seem to be precluded, for bromine, in contrast to iodine, is not poisonous in any of its com-

---

[1] *Cf.*, Berliner klinische Wochenschrift, 1896: Salubrol, ein neues antiseptisches Streupulver, von Dr. M. Silber, bish. Assistenzarzt an der kgl. Haupt-Klinik und im Fränkel'schen Hospital in Breslau.

pounds, and has therefore found very extended use as a therapeutic agent; and, although it cannot be denied that its continuous use for any great length of time seems undesirable by reason of bromine exanthema, nevertheless this incidental ill-effect is not to be compared with the serious and frequently alarming toxic phenomena which, as already mentioned, are liable to manifest themselves after the most moderate applications of iodoform.

The third advantage of salubrol is its considerable antiseptic power,—its efficiency as a germicide. Although, both in surgery and in dentistry, aseptic methods are aimed at so far as is practicable, yet in certain purulent septic processes it is sometimes impossible to avoid antiseptic treatment, and many operations require antisepsis, because it is not always practicable to keep the field of operation clear. Without entering in detail into Silber's bacteriological experiments,—for this I refer the reader to the above-mentioned treatise,—I must state briefly that his attempts at culture extended to five different species,—bacillus prodigiosus, pyocyaneus, tetragenus, staphylococcus, and anthrax. All these experiments render it sufficiently clear that salubrol has pronounced power as a germicide. Iodoform checks the growth of the bacteria, but does not kill them. Cultures that have been sprinkled with iodoform do not, it is true, continue to thrive, but when freed from the antiseptic and transferred to a fresh culture medium they resume growth. Salubrol, on the contrary, not only checks the growth of developing bacteria, but even kills off such cultures as are already well advanced.

Having had my attention called to these favorable experiments with salubrol, I began to test it in my own practice. I received a quantity of salubrol for experimental purposes from the manufacturers in Höchst, and I have been using this preparation for about two months, with an average of twenty cases daily. Without making a detailed report of the separate cases, I can affirm that I have made use of salubrol with the very best results wherever I was formerly accustomed to employ iodoform. To judge by my practical tests,—I did not have time for bacteriological investigations,—salubrol is in no respect inferior to iodoform; and after the experiments that I made with salubrol paste in cavities and root-canals that had not been thoroughly cleansed, allowing the paste to lie under a cap of gutta-percha for from three days to two weeks, I can readily understand that the new antiseptic must have a radical effect as a germicide, for otherwise pericementitis must have been the consequence in a majority of these tests. I have not been able, unfortunately, to obtain cases of pyorrhœa alveolaris for treatment during the last few months, otherwise I should have been glad to observe the action of salubrol in these cases also. Those treated by me with salubrol included pulpitis, the capping of pulps, the amputation of the pulp, the treatment of roots in all stages of inflammation, and root fillings. I was also able to establish satisfactorily the alleged power of salubrol as a styptic in a case of hemorrhage after the extraction of a lower second molar. The patient in this case, anæmic and hæmatophilic, had suffered from hemorrhage for days after every extraction. Accordingly, soon after the removal of the tooth I tamponed both alveoli with cotton saturated with salubrol, and was very agreeably surprised to find that the bleeding ceased at once and did not recur. It may readily be admitted that chance often played its part in my experiments; nevertheless, it seems to me, in view of the number of these tests, and in spite of the relatively short period of investigation (two months), that salubrol possesses antiseptic qualities in a high degree. I even believe that this agent, which seems to be in no way inferior to iodoform, but even surpasses the latter in value, and is, above all, free from its disagreeable properties, may be better adapted than any other to exclude iodoform entirely from the pharmacopœia of medicine and dentistry.

For practical use I have prepared salubrol in the following forms: as a paste of salubrol and arsenic, as a paste of salubrol and glycerin, and as a solution in alcohol, and also in chloroform. For purposes of injection I always prepare fresh liquid (aqua dest. 50, salubrol 5) by mixing the powder with a little water in a porcelain mortar, and then adding what water remains.

The paste of salubrol and arsenic (ac. arsen., 3; salubrol, cocaine, āā 1; ac. carbol., q. s. ut fiat pasta spissa) I consider very effective.

The paste of salubrol and glycerin is made by mixing the powder thoroughly with glycerin in a mortar, without further additions. I consider even the admixture of oxide of zinc for the capping of pulps as purposeless; the paste can be given more or less consistency as desired by the appropriate additions of salubrol powder. This paste keeps excellently in a closed glass; only it is necessary to stir it daily, as the salubrol in time rises to the surface. We see, therefore, that salubrol paste has the advantage over iodoform paste that it does not, like the latter, require further additions of the powder, which impair the action of the preparation,—*e.g.*, of the caolin or oxide of zinc used as a vehicle. The thick paste of salubrol always remains soft, and is therefore especially adapted to the capping of pulps and the filling of root-canals. In the case of decapitated pulps the necessary quantity of paste may be fixed on the desired spot with ease and certainty, and will remain there even if it should come in contact with saliva, as the preparation has no affinity for water. That salubrol remains permanently soft is obviously also an advantage as compared with the so-called cement pastes, for these last never serve the purposes of an antiseptic covering. Just because they gradually harden, they lose all antiseptic power. Only a covering that will remain at all times soft or even fluid is capable of protecting the pulp tissues or the remains of the pulp itself from further decay, and thus of acting permanently as an antiseptic mass.

The solutions of salubrol in alcohol and chloroform (saturated, and, so far as possible, always freshly prepared) serve chiefly to cleanse and dry the cavity before the salubrol paste is introduced. They are greatly superior to solutions of carbolic acid in that they have no eroding effect upon the adjoining mucous membranes. For this reason it is possible literally to flood a cavity with the alcoholic solution, or a root-canal with the chloroform solution, without having to apprehend any danger to the soft parts of the mouth, even if the patient should swallow some of the fluid. I prefer to use the solution of salubrol in chloroform for root-canals, both because chloroform will dissolve a larger quantity of the powder, and also because by the more rapid evaporation of this solvent (which may be promoted by blowing in warm air) the salubrol remains finely and evenly distributed on the walls of the canal.

My procedure in filling canals with the aid of salubrol is very simple. After having made a funnel-shaped enlargement of the orifice of the canal I first apply, in gangrenous or putrid cases, a small piece of caustic potash, and push this up gradually with a fine canal-probe as far as the foramen apicale. When the contents of the canal have been saponified, the entire canal is cleansed by means of a Pravaz syringe and subsequent thorough irrigation with lukewarm water; then with a flexible canal-drill, and without exerting any pressure, I enlarge the entire canal as far as the foramen, and in some cases even perforate the latter when there is suppuration or necrosis at the apex of the root. The canal is best dried by means of the chloroform solution of salubrol, introduced repeatedly on closely rolled spirals of cotton. After this the paste of salubrol and glycerin is gradually pumped in, until the canal is half full. The cavity is then closed with pink gutta-percha, and is left for several days, in some cases even longer, to give time for any reaction that may set in. Not until then is the final process of filling undertaken, in which the lower third of the canal is always left filled with as thick a paste of salubrol and glycerin as may be. The rest of the canal is then finished with Hill's stopping, or covered with a metal cap if the case is one for crown- and bridge-work. This metal cap, which is intended to receive the corresponding metal pin, is to be fastened down with phosphate cement, without allowing the cement to displace the salubrol paste, the latter, of course, being destined exclusively to close the foramen apicale.

